# Cronodent I, 3D interactive manual for self-learning of the dental chronology of equines

**DOI:** 10.3389/fvets.2025.1597710

**Published:** 2025-10-02

**Authors:** Evangelina Rodero, Ana González-Martínez, Andres Diz, Jesus Rodero

**Affiliations:** ^1^Department of Animal Production, University of Cordoba, Cordoba, Spain; ^2^Teaching Innovation Team 56-GID-UCO, Cordoba, Spain; ^3^Department of Anatomy and Comparative Pathological Anatomy, University of Cordoba, Cordoba, Spain; ^4^Department of Engineering, Synergya Tech, Cordoba, Spain

**Keywords:** 3D technology, equids, dental age, mobile app, self-study

## Abstract

One of a veterinarian’s routine tasks is animal identification, with age being a crucial factor in the identification and evaluation of individuals. Knowledge of the morphological changes in animal dentition used to determine their age is essential in veterinary education. Today’s students increasingly prefer innovative learning tools that incorporate 3D anatomical models over traditional 2D resources. As part of a teaching innovation project at the University of Córdoba, Spain, new educational materials were developed to aid students in identifying the age of animals using 3D images of equine incisor teeth. The main objectives of this initiative were to develop an app, Cronodent I, to support learning of equine dental chronology through 3D-scanned images of horses’ dental arches. A second objective was to evaluate students’ perception and acceptance of these new teaching materials, as well as their impact on academic performance. For this purpose, 17 equine dental arches were 3D scanned and incorporated into the Cronodent I app. The app’s usefulness was evaluated through a student satisfaction survey (*N* = 97) and, success rate and performance scores (*N* = 453). The app was easily accessible and received positive ratings for functionality and usability. The scores improved significatively (*p* < 0.05) from 4.37 to 4.86, following the success rate a similar trend: it increased more after the Cronodent I app became available to students on the Moodle platform (50.35% vs. 57.51%), but dropped to 48.76% when the app was accessible only on Android mobile devices. Cronodent I proved to be an effective educational tool for developing students’ skills in determining horse age based on dentition type and tooth wear stages. Cronodent I supports self-directed learning and offers mobile access to anatomical materials and resources, with accessibility emerging as a key factor in its overall success. Further research is needed to assess the long-term impact of Cronodent I on the learning outcomes of veterinary students to determine its effectiveness.

## 1 Introduction

Knowledge of the morphological changes that occur in animal dentition used to determine age is essential in veterinary education. This knowledge plays a critical role in individual identification checks and for handling registration documents related to ownership verification, marketing, veterinary forensics, and the transport of animals, particularly horses, are crucial. Usually, the age of the horse is determined based on changes in the incisors. These include differences in the shape and size of deciduous and permanent teeth, wear of the incisors (particularly on the occlusal surface), disappearance of the dental cup, appearance of dental stars, changes in the shape of the occlusal surface, presence of hook on the upper corner incisors, and the development of Galvayne’s groove, among others (1–3).

The European Association of Establishments for Veterinary Education (EAEVE), which in 2020 accredited the University of Cordoba School of Veterinary Medicine, advocates for the adoption of alternative methods to the use of live animals in student training (4).

The use of models for teaching human and animal anatomy dates back over a 1,000 years. During the 16th to 18th centuries, ivory was commonly used to craft models illustrating muscle structures and the position of major internal organs. In the 17th and 18th centuries, artists such as Ercole Lelli (1702–1766) used colored waxes to recreate highly realistic dissected figures and organs (5). The 19th century saw the introduction of papier-mâché anatomical models from France. One of the pioneers of this technique was Auzoux, who began developing anatomical models while in medical school, demonstrating great skill and passion for reproducing different organic systems (6). These models gained recognition for their versatility, usefulness, and remarkable realism (7).

With the purpose of teaching age identification in animals, Auzoux assembled a collection of dental arches from equids and bovines, which were acquired by various European veterinary schools, including the University of Cordoba (8). However, with continued use over time, these valuable pieces have deteriorated. Today, they are preserved as historical relics, being more than a century old. Later, bone specimens from slaughterhouses were used for teaching purposes, but these too degraded with repeated handling.

In recent years, the use of three-dimensional technologies, such as 3D digital models, 3D printing, and augmented reality (AR) or virtual reality (VR) environments, has gained significant relevance in the teaching of veterinary anatomy. These tools offer advantages over traditional methods based on cadavers or physical models, such as reduced ethical concerns, unlimited availability, educational consistency, and student motivation (9–12). Furthermore, scanned or printed 3D models significantly aid spatial understanding of complex anatomical structures, such as skulls or hearts in congenital pathologies, surpassing learning with 2D images alone (10).

The introduction of 3D modeling technologies offers a highly practical solution to the need for anatomical teaching materials. One of the most significant advantages of modern 3D measurement technologies is their ability to capture geometric aspects that are extremely difficult, or even impossible, to analyze using traditional methods (13). Portable 3D scanners can produce high-resolution, three-dimensional replicas, enabling the documentation, registration, and preservation of bone collections from any animal species (13).

A key advantage of 3D data acquisition is its cost-effectiveness in creating digital records of skeletal collections, which can be shared and accessed worldwide without the need for physical bone specimens. Furthermore, 3D images permit the subsequent creation of artificial anatomical models that can be easily replicated, measured, modified, and preserved (14). These physical replicas provide accurate and tangible representations of anatomical structures while maintaining the correct proportions, topographical relationships, morphological details, and color, without the risks of decomposition or contamination (15).

Additionally, this approach improves access to anatomical structures while addressing issues related to availability, biosafety, and ethical concerns, without compromising educational quality. The use of exact replicas offers a realistic learning experience and avoids the limitations of overly simplified or inaccurate anatomical models (16).

The use of 3D models in teaching offers the key advantage of easy reproducibility, ensuring long-term availability (17). Integrating these models into an app further enhances their durability, allowing for regular updates in line with technological advancements.

In the current educational paradigm, student-centered learning models are gaining importance, as they encourage active participation, engagement, and skill development (18). Today’s students, who have been highly exposed to information and communication technologies (ICT) throughout their lives, demonstrate a strong preference for innovative materials that incorporate 3D displays of anatomical structures, as opposed to traditional 2D resources. Studies have shown that this approach improves academic performance (19, 20). This shift in teaching strategies should motivate instructors to integrate new materials and teaching techniques, facilitating the transition from theoretical knowledge to practical application from anatomical replicas to real-life recognition of structures in animals, focusing on solving real-world veterinary problems. Furthermore, the acceptance by veterinary students of 3D models as a complement to the use of cadavers and plastinated material is high (21).

As part of the University of Cordoba’s Teaching Innovation Project (2022–5-3003), new educational materials were developed to assist veterinary students in identifying the age of animals using 3D images of equine incisor teeth. This knowledge can be applied to veterinary inspections of both living and deceased equids.

The application presented and evaluated here was made available to students in advance, ensuring that those enrolled in the Ethnology, Ethology, and Animal Welfare course, taught in the second semester of the first year of the Bachelor’s Degree in Veterinary Medicine, had access to it from the beginning of the semester. Currently, dental chronology in horses is taught in this course by means of explanations supported by 2D images of dental arches projected on a screen to illustrate the criteria for identifying dental age. Additionally, students are provided with bone models of dental arches in class to facilitate the learning process. However, today’s students differ from those of previous decades, as they are accustomed to working with ICT tools. This shift was a key motivation for developing Cronodent I, which builds on the existing bone models that were deteriorating, as mentioned above.

The Cronodent I app was developed to contribute to the following learning outcomes (degree training competencies): (i) understanding morphology and organ/system topography and their applications; (ii) knowledge of ethnological and productive characteristics, with a special focus on management and its application; (iii) knowledge of the principles of ante- and post-mortem veterinary inspection and their applications; (iv) practical application of veterinary medicine principles and methodologies, as well as the acquisition of the skills and competencies outlined in the degree program’s general objectives.

Moreover, considering the health, ethical, and financial challenges associated with cadaveric dissections, the use of cadavers has declined, creating a gap in 3D learning, especially in fields like anatomy. As our results indicate, students found Cronodent I to be user-friendly, which was one of the initial objectives. The app was also designed to be a modern learning tool aligned with students’ current interests and motivations, as well as the new space–time paradigm for study habits. Nevertheless, it is not intended to replace face-to-face classes; rather, it serves as a supplementary tool that allows students to access bone models at any time and self-assess their learning.

The main objectives of this initiative were: (i) to develop an interactive manual for equine age identification, including a mobile app for learning dental chronology in equids using 3D-scanned images of horses’ mouths; (ii) to improve the available teaching resources and enhance the quality of veterinary education by promoting self-learning in equine age recognition through dentition; (iii) to assess students’ perceptions and acceptance of the new teaching materials; and (iv) to evaluate improvements in students’ academic performance in identifying dental age.

## 2 Materials and methods

### 2.1 Selection of anatomical material

A meticulous selection process was carried out to determine the dental arches to be scanned, choosing from all the natural anatomical pieces of equid incisors available in the Department of Animal Production and Department of Animal Anatomy at the University of Cordoba. Selection criteria were based on type of dentition and chronological stage. In total, nine complete dental arches (upper and lower) from equids were selected. They were selected to represent a wide range of ages (from 16 months to 18 years) and various stages of wear (worn, erupting, and leveled). The app currently includes dental arches with deciduous teeth (2), permanent teeth (5), and a combination of both (2), each displaying different stages of incisor wear.

### 2.2 3D scanning procedure

The bone samples were 3D scanned using the Artec Spider, a handheld high-resolution 3D scanner that employs blue structured light technology to capture objects with exceptional detail and accuracy. This scanner is particularly well-suited for small objects or highly detailed and intricate areas of larger objects. The Artec Spider offers a resolution of 0.05 mm, allowing it to capture even the finest details with high accuracy. It also scans objects in full color, providing realistic and detailed texture information.

Data acquisition and processing were performed using Artec Studio r13 software. The results were exported in a standard 3D file format (*.obj) compatible with any API integrated into a platform or app. These high-fidelity 3D digital images are unalterable and can also be used to produce 3D-printed models in durable plastic materials for practical classroom applications.

**Figure 1:**
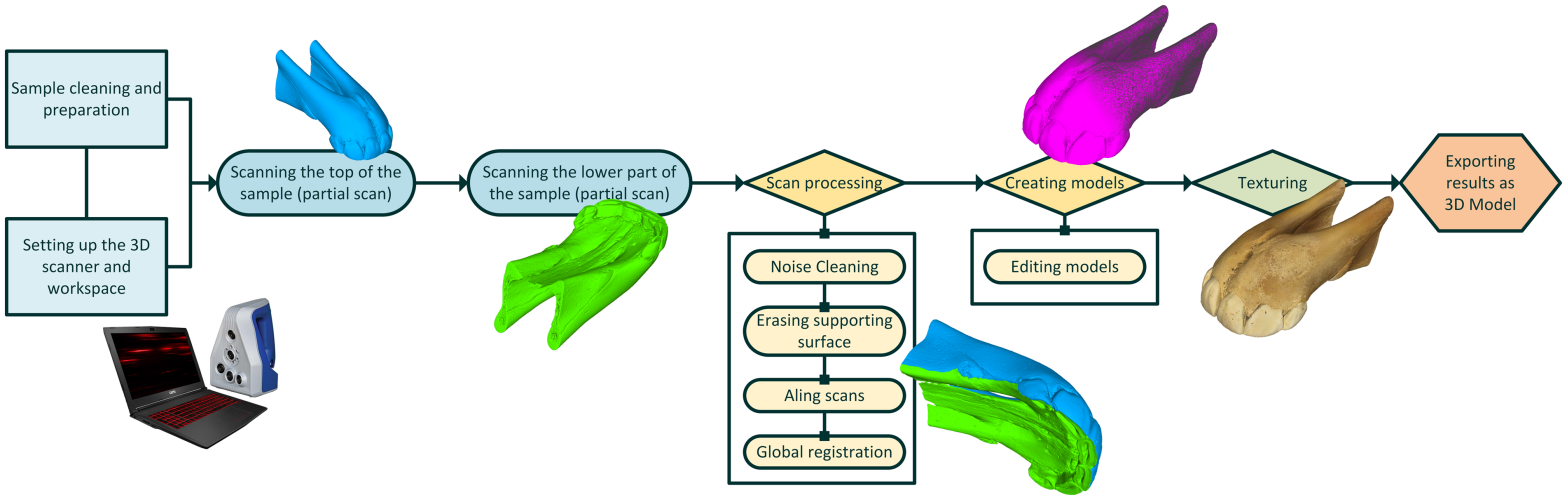
Illustration for scanning setup and procedure.

**Figure 2:**
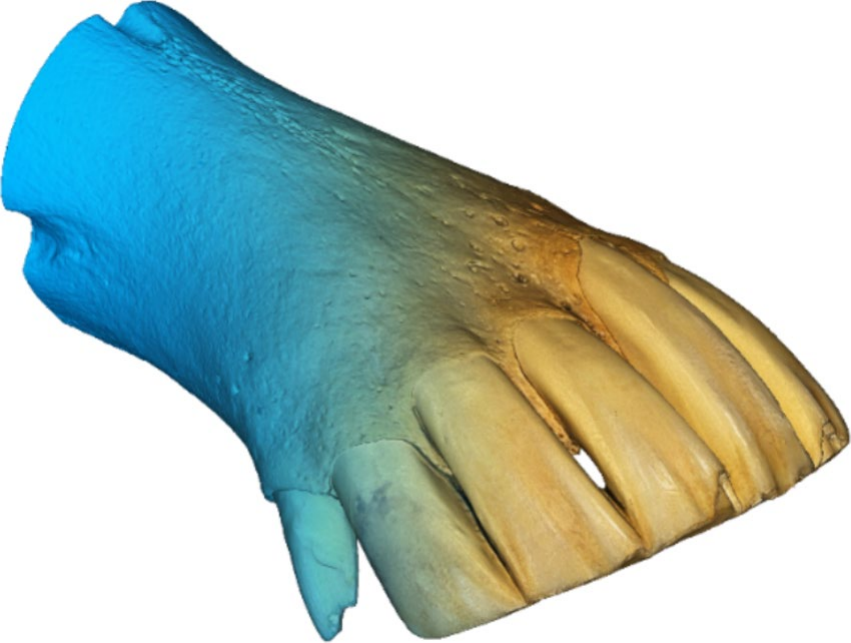
Result of the 3D scanning process, polygonal mesh, and textured model of lower dental arch no. 7.

**Figure 3:**
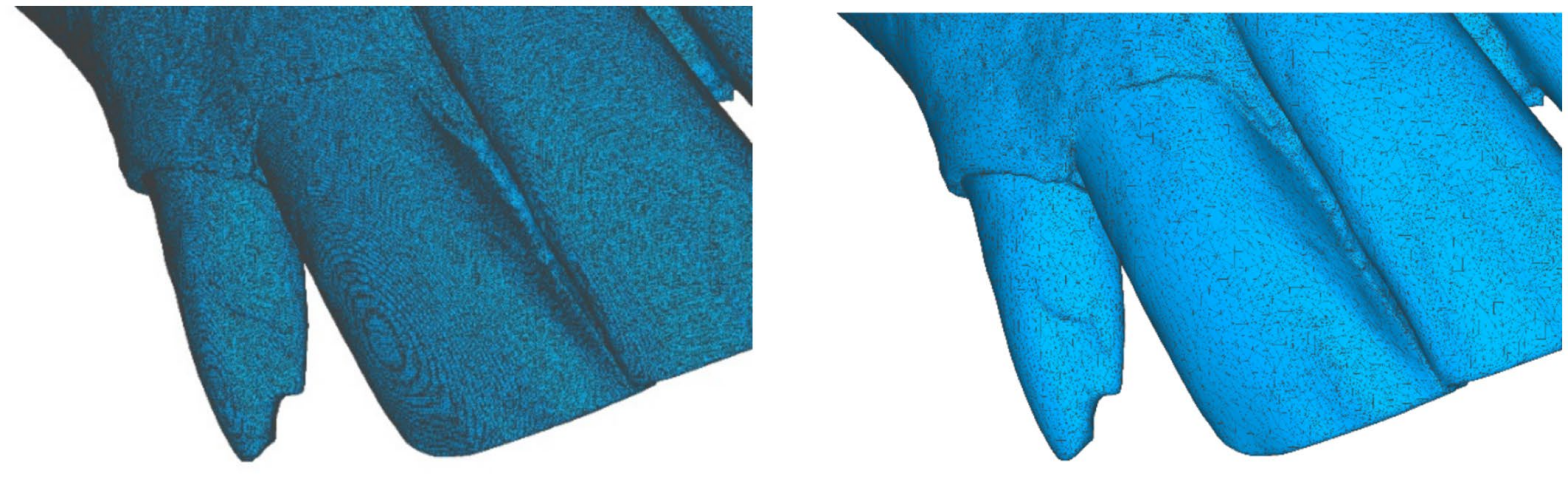
Polygonal density comparison between the original high-resolution 3D model (left) and the 3D mesh after the optimization process (right).

**Figure 4:**
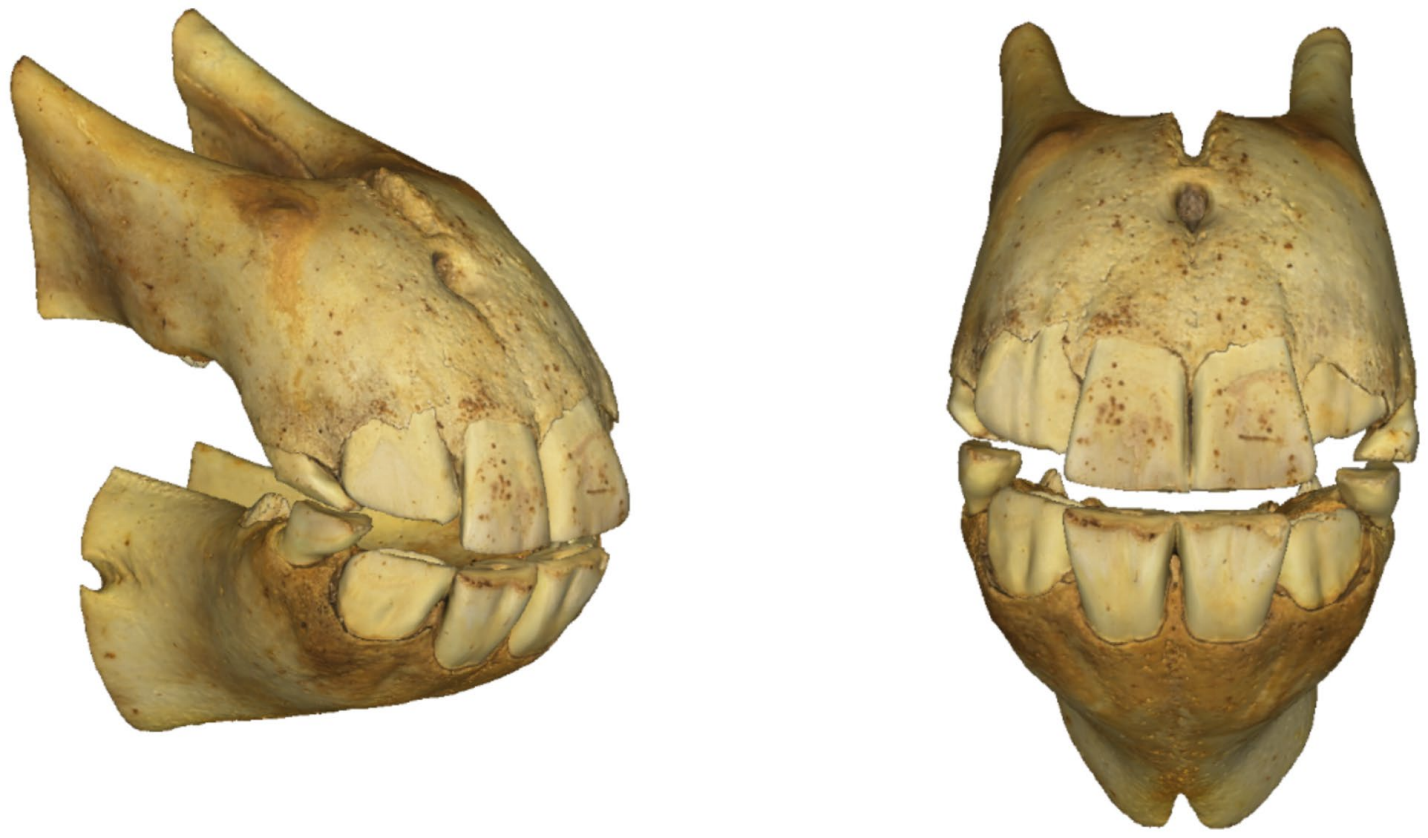
Virtual emplacement of the 3D models of the upper and lower arches.

The final 3D models were obtained by performing partial scans of each dental arch, which were then merged using processing software through a procedure known as scan registration. Unwanted elements and noise were filtered out, and a high-resolution polygonal model was generated. Finally, realistic textures were applied using high-resolution photographs captured by the 3D scanner (Figures 1–4).

A total of 29 3D models of dental arches were obtained, corresponding to both upper and lower arches. Initially, each scanned model contained an average of 6.5 million polygons, resulting in a file size of approximately 300 MB. This file size was too large for inclusion in a mobile-oriented app. To optimize the models, polygonal mesh decimation was applied using a precision-based mesh optimization technique. This reduced the models to an average of 85,000 polygons (approximately 5 MB), representing just 1.3% of the original polygon count and 2% of the original file size. The final optimized file size was suitable for mobile app implementation, with no noticeable loss of detail.

Only nine complete dental arches (upper and lower) were included in the first prototype of Cronodent I. This preliminary version, accessible only from a mobile or tablet with and android system, was evaluated for its usability and impact on student learning outcomes. Because not all students had access to the first version of Cronodent I due to not owning a mobile device with Android, we decided to develop a new version. The second prototype, which was provided to all the students via Moodle, expanded the collection to 17 dental arches, though some were incomplete pairs of upper and lower arches.

### 2.3 App Cronodent development

**Figure 5:**
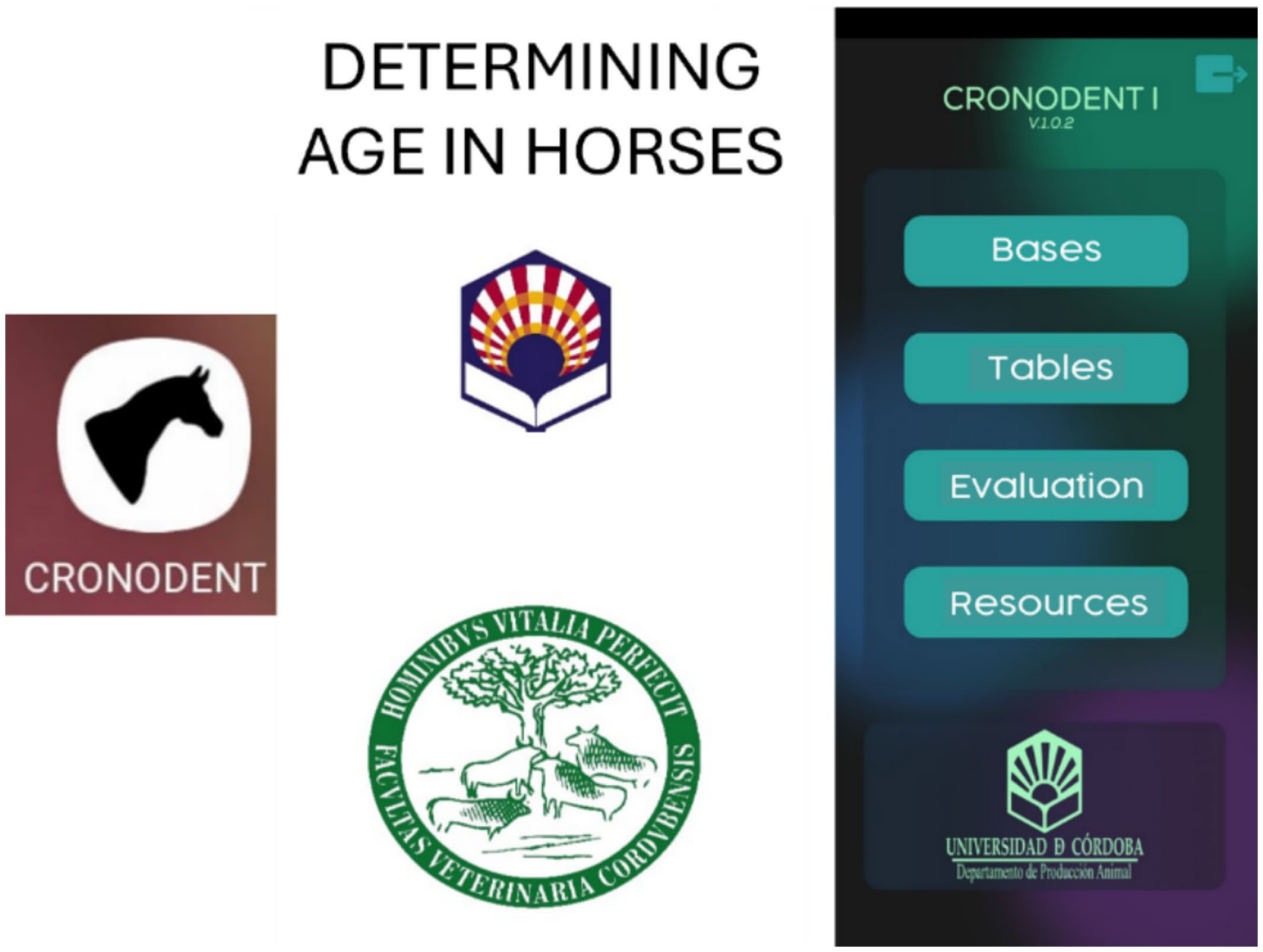
Access to Cronodent I for determining age in horses (left) and main window (right). Reproduced with permission.

The Cronodent I app is available for Android mobile phones and tablets (Figure 5). Developed in collaboration with the CETEMET Technological Centre, the app features interactive 3D models that users can rotate and manipulate, simulating the experience of holding a real dental arch in their hand. While the app is currently free to download, access to the evaluation module is restricted to students at the University of Cordoba who are enrolled in the subject of Ethnology, Ethology and Animal Welfare.

Cronodent I was designed with an expandable 3D image bank, allowing new models to be added periodically. This feature enables students to enhance their learning with an increasing number of examples. The app also includes an evaluation module that allows students to assess their progress in self-learning.

**Figure 6:**
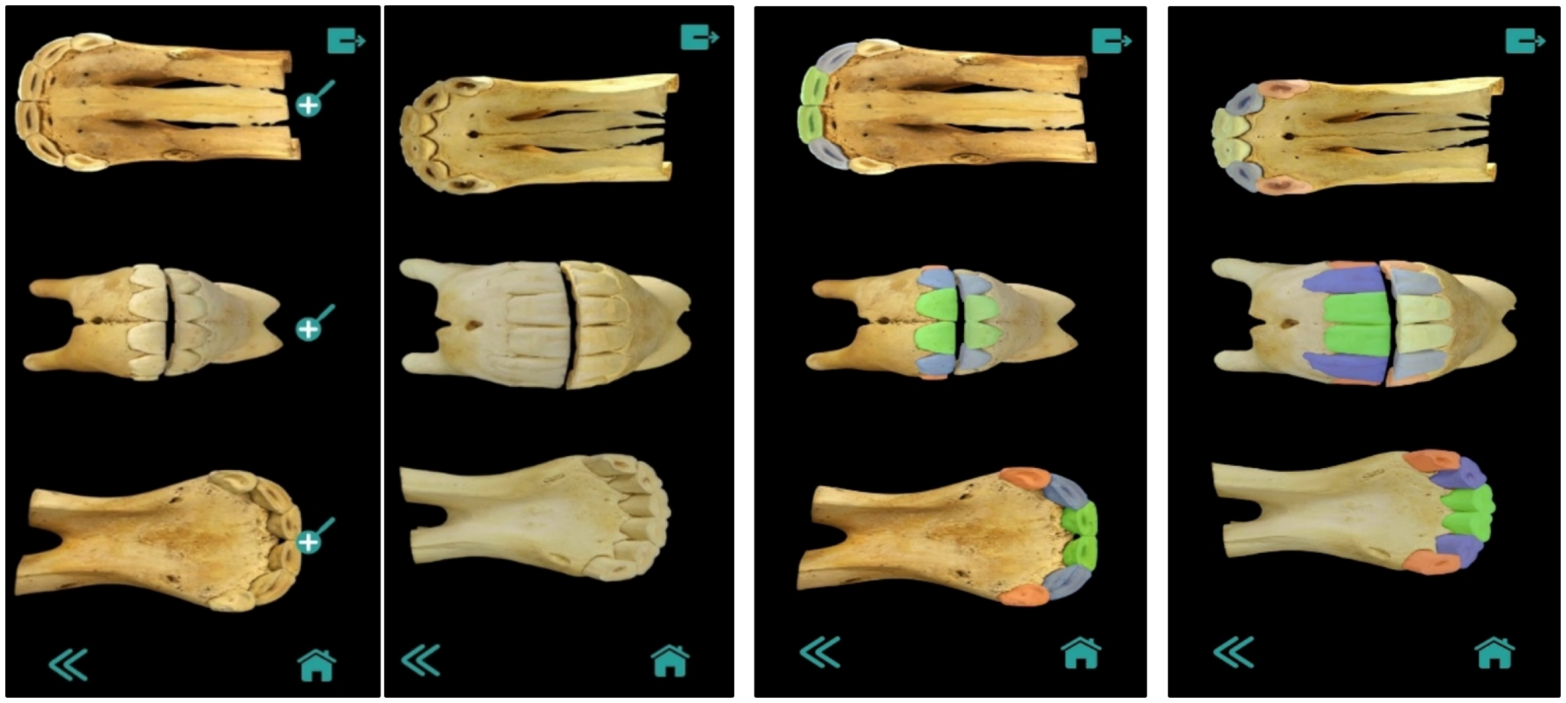
Images of dental bases for determining age in horses (deciduous and permanent teeth and order of eruption).

The main window of the application, accessible from any point in the app via the “Home” button, displays the four main options: Bases, Tables, Evaluation, and Resources (Figure 5). The “Bases” window provides access to the application’s core resources, which provide the information needed for later self-assessment. These resources are organized into four sections: (i) types of teeth (primary, permanent, or a combination of both); (ii) eruption order (a looping GIF that illustrates the sequence of the process with color-coded dental pieces); (iii) wear level (worn, abraded, and leveled); and (iv) wear order (a looping GIF that illustrates the sequence of the process with color-coded dental pieces) (Figure 6).

**Figure 7:**
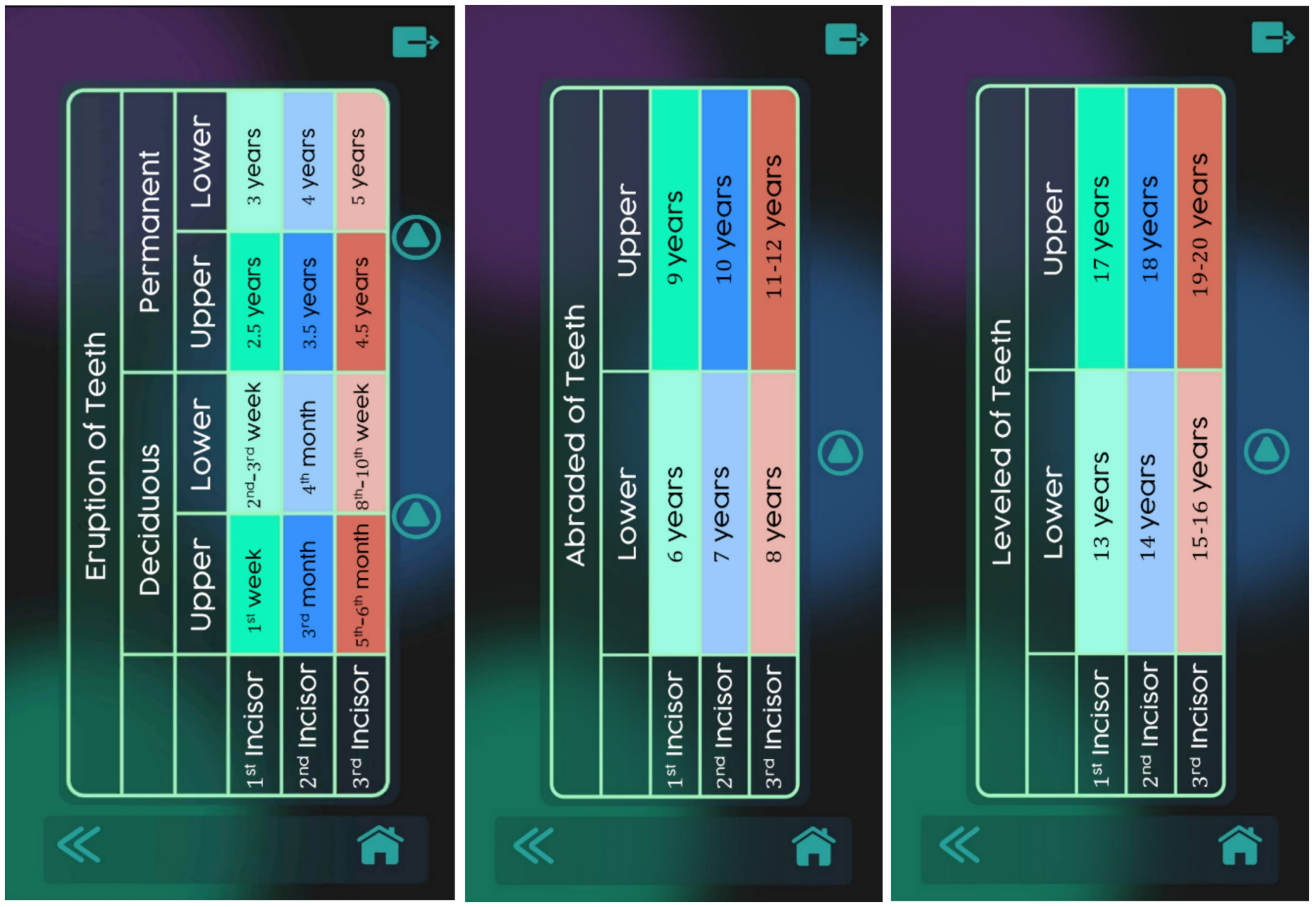
Tables to determine horse age (eruption and wear) in the Cronodent I app. Reproduced with permission.

The “Tables” window includes representative tables indicating the typical ages at which the different stages of each process (eruption, wear, and leveling) occur, each marked with a distinctive color (Figure 7). For each of the three processes (with both deciduous and permanent teeth are included in the eruption tables), a button allows users to view the corresponding GIF of the process, with the stages of eruption, wear, and leveling identified by colors.

**Figure 8:**
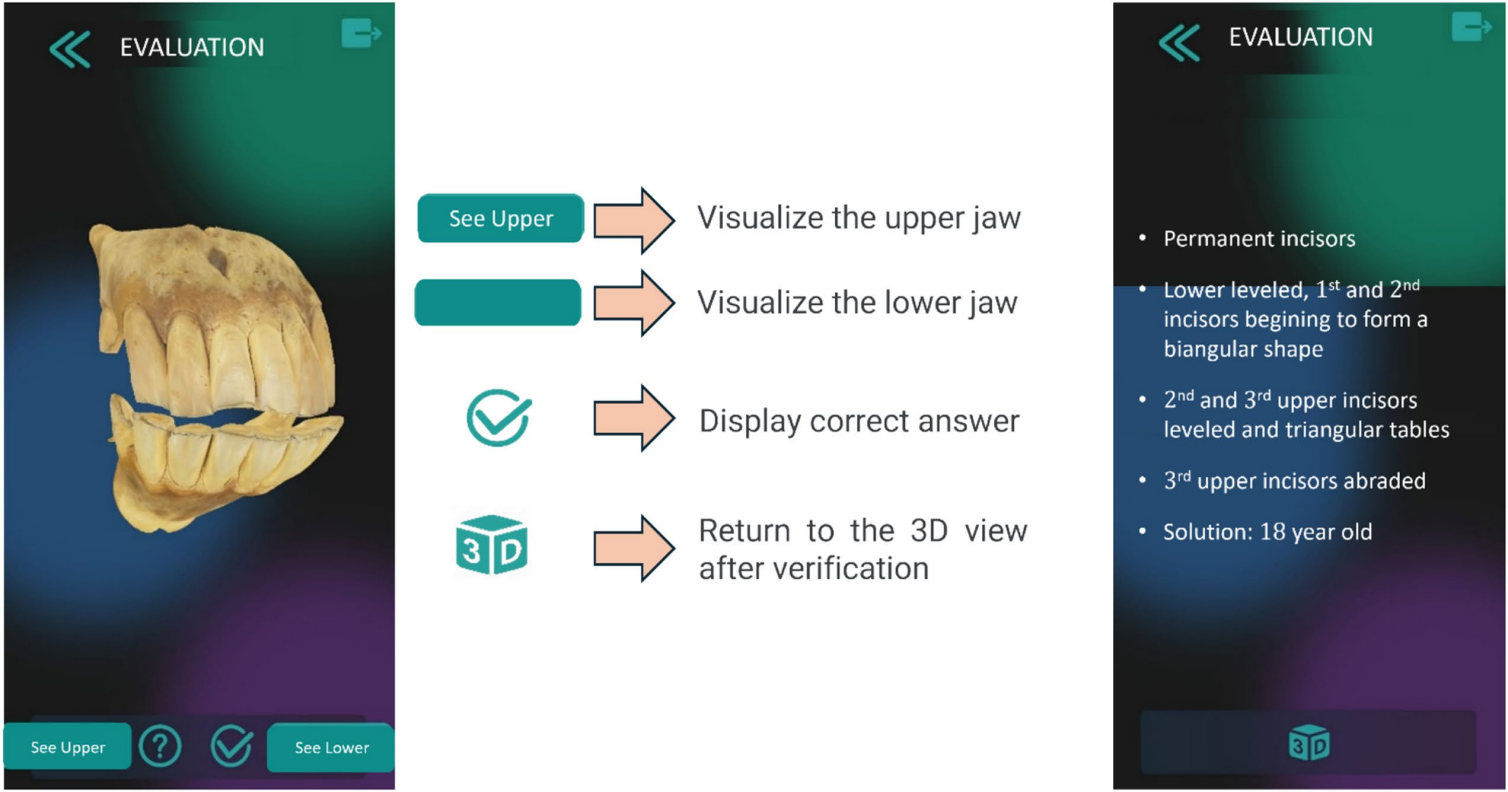
Self-evaluation module in the Cronodent I app. Reproduced with permission.

The “Evaluation” window enables self-assessment by allowing users to identify characteristics of 10 randomly generated 3D models, which can be controlled using the buttons shown in Figure 8. Touch controls allow users to zoom in and rotate the models. The “Resources” window provides access to a set of Supplementary material on equine dental chronology. These materials include not only the schematic information presented in the app, but also additional criteria for determining the age of horse based on its dental arches. The criteria include changes in the shape of the occlusal surface; disappearance of the infundibulum (“cup”) and permanence of the enamel ring; the shape, presence, and position of the dental star; Galvayne’s groove; the presence of a hook on the upper corner incisors, and changes in the profile of the upper and lower dental arches, among others.

Additionally, all the windows in the app include a set of general-purpose buttons that perform the following functions: (i) return to the previous window (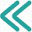); (ii) display or hide a brief explanatory text about the current window (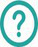); (iii) navigate to the application’s main menu (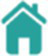); (iv) immediately close the application (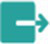).

### 2.4 Evaluation of students’ opinions regarding the Cronodent I application

A survey consisting of eight questions with Likert-type responses was developed to evaluate users’ perceptions and experiences with Cronodent I (see Supplementary material). All items were measured on a Likert scale ranging from 1 (*strongly disagree*) to 6 (*strongly agree*). This forced-choice format, without a neutral midpoint, was chosen to encourage respondents to express a clear preference and avoid central tendency bias. The scale items were presented directly in the questionnaire, and participants selected a single numerical value to reflect their opinion. This type of ordinal scale is well-suited to the context of veterinary education, as it enables the collection of standardized yet nuanced responses regarding students’ perceptions of educational tools, such as 3D anatomical models, while facilitating the application of non-parametric statistical analyses.

To assess the adequacy of the sample size, the Kaiser–Meyer–Olkin (KMO) and Barlett tests were performed. A KMO value above 0.50 and the probability below 0.05 indicate that the data are suitable for factor analysis (22). Additionally, Cronbach’s alpha was calculated to assess internal consistency, with reliability analysis conducted on selected variables. Each achieved a score above 0.75, indicating acceptable reliability (23).

The survey was conducted in the 2022–2023 academic year among first-year students in the Bachelor’s Degree in Veterinary Science at the University of Cordoba. A total of 72 of the 150 students enrolled in the Ethnology, Ethology, and Animal Welfare course completed voluntary the survey. At that time, the app was only available on Android mobile devices. The survey was repeated in the 2023–2024 academic year, when the app became available on the Moodle platform, and was completed by 25 students. The students did not receive any incentive for their participation and the survey was distributed on paper by the teachers of the Ethnology, Ethology, and Animal Welfare subject during the last class to maintain the anonymity of participants.

### 2.5 Analysis of improvement in learning outcomes with the incorporation of Cronodent I in teaching

One of the thematic units in the Ethnology, Ethology, and Animal Welfare course focuses on identifying horse age; an assessment traditionally performed for over 50 years using 2D photographic references. Final exam results from the past three academic years were compared to evaluate the effectiveness of Cronodent I in enhancing student learning in this area, final exam results from the past three academic years were compared. The analysis considered both the grades obtained in the section related to horse age identification, as well as the success rate, defined as the number of students who passed that part of the exam.

The comparison incudes data from three academic years: one prior to the development of the app (the control year, 2021–2022), and two following its implementation (2022–2023 and 2023–2024). Analysis of variance (ANOVA) and Tukey *post hoc* tests were performed to identify statistically significant differences in student scores across the three academic years. Statistical significance was set at *p* < 0.05. Survey data and student scores were analyzed using Microsoft Excel^®^ for Windows and Statistica 12.0 software.

## 3 Results

**Figure 9:**
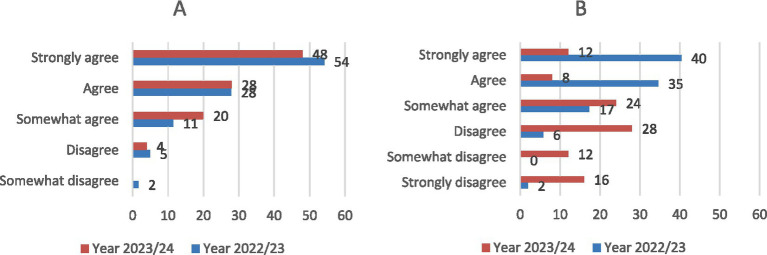
Student opinion results (*N* = 97) on accessibility or localization of the app **(A)** and correct functioning **(B)**.

The results of the satisfaction survey showed that, in the first academic year (2022–2023), 66.2% of the 72 participating students voluntarily used the Cronodent I tool, which was only accessible to those with Android mobile devices. In the following academic year (2023–2024), when the app was made available to all students through the Moodle platform, voluntary use increased significantly to 96% among the 25 students surveyed. When asked whether the application was easy to locate in the Moodle platform, 93.4% (2022/23 academic year) and 96% (2023/24 academic year) of respondents found it accessible (Figure 9A). Regarding functionality, 92.3% of students reported that the app worked correctly. This percentage dropped to 44% in the subsequent year (Figure 9B).

**Figure 10:**
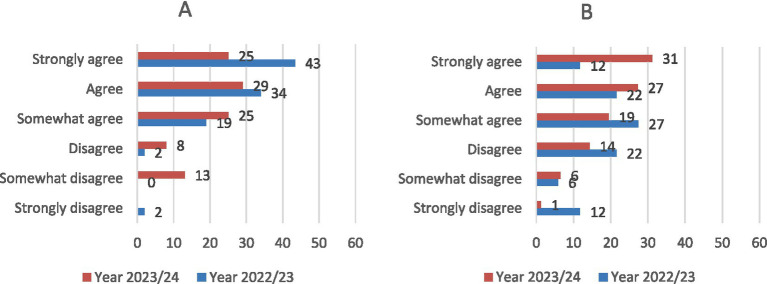
Student opinion results (*N* = 97) regarding the app’s interface usability **(A)** and frequency of use **(B)**.

**Figure 11:**
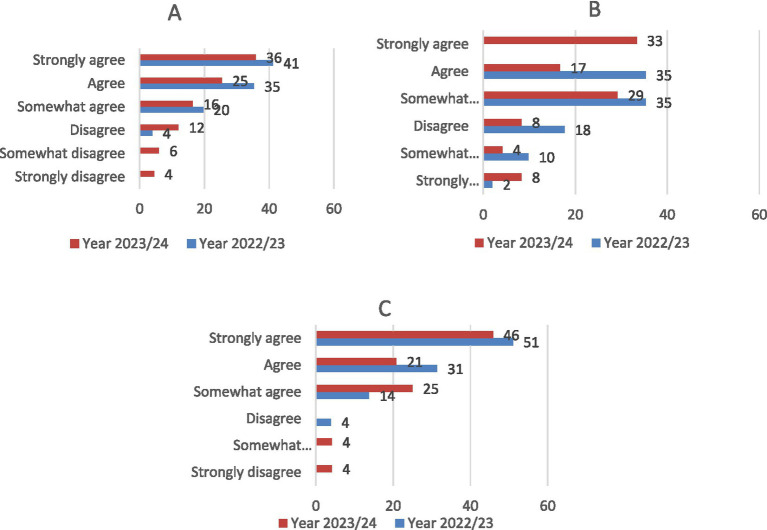
Student opinion results (*N* = 97) on its effectiveness in equine age determination **(A)**, identifying wear stages **(B)** and types of dentition **(C)**.

Regarding the app’s interface usability, 96.2% of students indicated that the interface was user-friendly and easy to navigate, with 60.8% reporting frequent use (Figure 10). Students also considered the app an effective teaching tool for learning how to determine the age of horses (Figure 11A). Furthermore, 88.2 and 96.1% of users confirmed the Cronodent I app’s effectiveness in identifying the stages of dental wear and type of dentition in equines, respectively (Figures 11B,C). Students who were enrolled in the Ethnology, Ethology, and Animal Welfare course in the following academic year (2023–2024) reported lower levels of agreement regarding the usability, functionality, and effectiveness of Cronodent I.

The evaluation of students’ ability to determine an animal’s age based on dental arches showed significant improvement (Table 1). Students not only rated the Cronodent I app positively but also achieved better outcomes. Their mean scores improved significatively (*p* < 0.05) from 4.37 out of 10 in the 2021–2022 academic year to 4.86 in the 2023–2024 academic year. However, in the year when the mobile equine age determination tool was first introduced, the mean scores decreased significantly (*p* < 0.05) to 4.23. The proportion of students who obtained a score of 5 or higher in the determination of dental age in horse was similar in the pre-Cronodent I academic year and when the app was provided solely through Android mobile devices (50.71 and 49.07%, respectively). However, this data improved substantially in the 2023–2024 academic year, reaching 57.89%, when the Cronodent I app was facilitated to the student via Moodle. The success rate showed a similar trend: it increased more after the Cronodent I app became available to students on the Moodle platform (50.35% vs. 57.51%), but dropped to 48.76% when the app was accessible only on Android mobile devices.

**Table 1:** Students’ assessment results for equine age determination based on dental arches over three academic years.

		Number of observations	Success rate on the first test (% students passing out of the total tested)	Score (mean)	Score (standard deviation)
Year	2021–2022^1^	140	50.35	4.37^ab^	2.10
	2022–2023^2^	161	48.76	4.23^a^	2.31
	2023–2024^3^	152	57.51	4.86^b^	2.40
*p*-value				0.04	

^1^Pre-Cronodent; ^2^Post-Cronodent I (nine 3D models); ^3^Post-Cronodent I (seventeen 3D models); ^a,b^ Different letters indicate statistically significant differences at *p* < 0.05.

## 4 Discussion

Integrating new teaching methods and modern technology fosters student engagement and knowledge acquisition (24). In recent years, technology has increasingly replaced traditional teaching methods with visually rich, interactive 3D computer-generated images (17). Unlike conventional 2D images, which are flat and depict only length and width, 3D models add depth, making them more realistic (25). There is strong evidence supporting the effectiveness of educational apps in learning, particularly when used alongside existing methods (26).

Learning involves transforming information into knowledge derived from experiences and sensory input, such as sight, hearing, and touch. The brain encodes and stores information through knowledge processing (27). Visual material has proven to be a highly effective teaching tool, with studies showing that it improves memory retention compared to traditional methods (17). Research has shown that students “learn more, remember longer, and show more interest in learning when training films are used” (28). Experiential learning, which emphasizes concrete experience, reflection, and experimentation, has also been shown to accelerate learning (29).

### 4.1 Adaptation of the Cronodent I app design for learning how to determine the dental age of equines

The Cronodent I app is structured as an illustrated guide, showing the different changes in horse’s teeth over time (eruption of deciduous and permanent dentition, wear, leveling, etc.) and in the dental arches (i.e., profile), relating them to the horse’s age. It is designed to periodically incorporate new 3D models, allowing students to expand their knowledge with a greater variety of examples. Furthermore, the 3D manual aims to integrate applied morphology with cutting-edge technological resources for educational purposes, searching in equine age determination while maintaining its relevance over time. Additionally, it seeks to show that new three-dimensional object reconstruction techniques are valuable for acquiring the skills and competencies required from the first day of a veterinary professional’s career.

Additionally, the interactive labels promote guided self-learning, in accordance with constructivist approaches. The app also incorporates commonly used icons that follow established design conventions, thus reducing cognitive load and facilitating intuitive navigation. By leveraging interface elements that students are already familiar with from other digital learning tools, the design supports smoother interaction and minimizes the time required to learn how to use the app. This is in line with principles of user-centered instructional design, promoting accessibility, and reducing extraneous cognitive processing.

The images in the Cronodent I app were scanned using high-resolution, portable 3D equipment. Unlike computed tomography (CT), this technology does not penetrate tissues, resulting in highly realistic representations of the external surface of the incisors at a lower cost. It is important to note that dental age is determined by examining the external characteristics of the teeth, making it unnecessary to view internal structures.

Various tools are used in teaching anatomy in veterinary medicine, ranging from atlases and apps to 3D models made of different materials, such as bone, plastic, or printed components. Among the more modern tools for identifying dental age is Horse Dental (Equine Dental Clinic), which presents a series of images highlighting key aspects of dental age identification. Another resource is Equine Dentistry Learning Modules, a set of virtual modules with interactive 3D models developed by veterinary schools such as those of the University of Guelph and Colorado State University.

Additionally, the “CHAP—Horse Aging Service” app allows users to determine a horse’s age from a photo of its dental arches. However, unlike the previous tools, it is not designed solely for educational purposes but can be used by the general public (30).

Each tool is useful for training future veterinarians. However, the ability to create 3D models based on real dental arch bones not only gives new life to existing material but also allows the 3D printing of models to replace worn or damaged specimens. At the present time, Cronodent I is only available on the Android platform, which is the most widely used platform in Spain. Nonetheless, efforts are underway to develop an iOS version. In the meantime, to ensure accessibility for all students, Cronodent I has been integrated into the University of Cordoba’s virtual classroom (Moodle) and is currently available to students enrolled in the Enology, Ethology, and Animal Welfare course.

As mobile and tablet devices become more widespread, there is a global trend toward using them as learning tools. In recent years, 3D applications have gained popularity by providing more effective practical training (31). Augmented reality (AR) is also gaining traction as an educational tool across various fields, enhancing students’ performance and learning experiences due to its interactivity and visual appeal (32). However, research attempting to prove its benefits in learning 3D geometry has been inconclusive due to methodological limitations, such as insufficient evaluation of user experience (32). A recent study found that students using a 3D application in their learning process could observe images much more effectively than in 2D textbook figures, as they were able to rotate and examine fine details (31). Moreover, the ability to access the 3D app at any time after classroom instruction increases engagement, satisfaction, and competency in understanding the subject matter (31).

### 4.2 Student perception of Cronodent I

According to our results, Cronodent I was easy to use, which was one of the app’s primary initial objectives. Based on the student survey, accessibility was also rated positively by over 93% of respondents, highlighting its importance as a contributing factor. Student perception for usability of Cronodent I received higher ratings when Cronodent I was made accessible via Moodle in the 2023–2024 academic year, being similar to those reported for other educational tools in veterinary teaching that incorporate 3D organ models (33).

Students satisfaction, evaluated through the question on perceived learning effectiveness, showed favorable results, consistent with findings from other 3D computational models used in veterinary education (21, 34).

The ability to rotate 3D models and explore anatomical structures supports both visual and kinesthetic learning styles, facilitating spatial understanding in line with the principles of multimodal learning (39). Cronodent I introduces a kinesthetic component of rotation and image manipulation that stimulates mental structures.

### 4.3 Improvement of Cronodent I as a learning tool for determining dental age in equids

A study found that students perceive AR and 3D models, such as those integrated into the EduCITY application, innovative and helpful in facilitating understanding, particularly in subjects like biology and plant species identification (35). In our case, the significant improvement in academic performance of veterinary students in the Ethnology, Ethology, and Animal Welfare course, particularly their ability to accurately determine equine age from dental arches, shows the practical usefulness of the Cronodent I app in learning equine dental chronology. Similarly to those observed by Schirone et al. (11), the survey results demonstrate a greater motivation by students.

A review of the use of 3D anatomy models in education found no conclusive evidence that students who use advanced models perform better than those who learn with traditional methods. This highlights the need to evaluate both short- and long-term outcomes (36). Cronodent I was evaluated over a short period, as it was introduced during the 2022–2023 academic year, which allowed for comparison of academic performance across only two academic years. Despite this limited timeframe, clear improvements were observed in students’ ability to identify the age of horses using 2D images.

It is also worth noting that Cronodent I initially included only nine 3D models. However, it was designed with the capacity for gradual expansion of anatomical content. For the 2023–2024 and 2024–2025 academic years, the app included 17 anatomical model (an increase of 8 models) covering ages from 14 months to 15 years. This expanded content provides students with a broader range of resources to understand the changes that occur in dental arches as horse age.

The primary objective for developing Cronodent I was improve the poor academic performance of students in determining horse age. In many cases, students showed a lack of interest in the subject, often underestimating its importance in everyday veterinary practice. Although dental arch bone specimens have always been available, students frequently did not use them, whether due to time constraints or low motivation, leading to a gradual decline in their use as a tool for reinforcing equine aging knowledge.

This situation inspired the development of an app that allows 3D models to be accessed anytime, from any mobile device. The success rate and scores improved after the Cronodent I app was made available to all students. This improvement was evident when the tool was accessible through Moodle, not just to those with Android mobile devices. Therefore, accessibility proved to be a key factor in the app’s success.

Cronodent I has improved the learning process for identifying the age of horses based on their dental arches in terms of both the student pass rate and the average score obtained. However, the results of this study suggest that, in addition to accessibility via Moodle, the increase in the number of images included in the app was also a significant factor contributing to the outcomes.

Familiarity with the new tool throughout the courses may also represent a factor to consider, as the student sample from the 2023–2024 academic year was not standardized in terms of prior knowledge of the tool. Students from the 2022–2023 academic year that were already familiar with Cronodent I may have interacted with the 2023–2024 students, potentially influencing subsequent evaluations. The results of this study are preliminary; a longitudinal assessment should be conducted by standardizing both the sample conditions and the features of the application itself. These limitations have been noted in several studies employing 3D tools in the teaching of medical disciplines (37).

### 4.4 Limitations

Given these improvements, we believe Cronodent I is a valuable tool for veterinary students to learn equine age identification remotely. It eliminates dependence on physical dental arch specimens, which have deteriorated over the past 40 years, and offers unlimited use without degradation over time. Additionally, it supports 3D printing of the scanned specimens.

However, one limitation is the need for students to become familiar with the tool. It is not intended to replace traditional classroom teaching, but rather to serve as a complementary learning resource. The app promotes self-directed learning and provides visual access to anatomical materials via a mobile device. Nonetheless, improvements are needed to ensure compatibility with other operating systems in addition to Android.

Additional enhancements could include providing instructors feedback based on app usage data—particularly from the assessment module (e.g., number of correct answers and attempts). Expanding the 3D image database would also increase the test variability and allow for the incorporation of additional features relevant to equine age determination.

Finally, one limitation to consider is potential sampling bias: less motivated students or those struggling to understand the content may have been underrepresented because the use of the app and participation in surveys was voluntary, which could have influenced the observed success rates. Differences in the mode of access to the app among the students in the sample (only students with Android devices vs. all students via Moodle) may have influenced the lower usability and satisfaction ratings given by students in the 2023/24 academic year, despite the observed improvement in performance. Student preferences regarding the use of mobile applications for learning purposes could account for these differences (38). Consequently, those who responded to the survey in 2023/24 may have had a more negative perception of the app’s usefulness as a learning tool.

## 5 Conclusion

The Cronodent I app provides effective and relevant teaching material for developing skills in determining a horse’s age based on the dentition type and tooth wear stages. These skills are transferable to real-world situations requiring the identification and assessment of both live and deceased horses.

Further research is needed to assess the long-term impact of Cronodent I on the learning outcomes of veterinary students to determine its effectiveness.

Student feedback and academic performance results suggest that Cronodent I is an appealing tool for veterinary students to learn how to identify the age of horses based on their dental arches. However, further efforts are needed to enhance its academic effectiveness and encourage broader student adoption.

Improvements in a new version of Cronodent I, such as diversifying accessibility, expanding the image database and incorporating add-ons that provide feedback to instructors, may be worthwhile to enhance its educational value and effectiveness. These additions could include data on app usage and self-assessment results, helping teachers identify the most challenging topics for students and areas in need of further development.

## Data Availability

The raw data supporting the conclusions of this article will be made available by the authors, without undue reservation.
