# Supplementary material for: Cronodent I, 3D interactive manual for self-learning of the dental chronology of equines
Source: Front Vet Sci. 2025 Oct 2;12:1597710. doi: 10.3389/fvets.2025.1597710 (PMC12529936; doi:10.3389/fvets.2025.1597710)
Supplement: Supplementary file 1 [file Table_1.docx]

Supplementary Material

**STUDENT OPINION SURVEY ON THE CRONODENT I APP DESIGNED WITHIN THE TEACHING INNOVATION PROJECT FROM UNVERSITY OF CORDOBA, SPAIN**

**Instructions**

Choose the option that best reflects your opinion:

1: Strongly disagree

2: Disagree

3: Somewhat disagree

4: Somewhat agree

5: Agree

6: Strongly agree

If you do not have sufficient criteria to evaluate an item, you may leave it blank

1. Have you used the application developed for determining dental chronology in equines?

| - Si |  | - No |  |
| --- | --- | --- | --- |

1. The application was easy to locate within the "Moodle" environment

| - 1 |  | - 2 |  | - 3 |  | - 4 |  | - 5 |  | - 6 |  |
| --- | --- | --- | --- | --- | --- | --- | --- | --- | --- | --- | --- |

1. The tool functioned correctly

| - 1 |  | - 2 |  | - 3 |  | - 4 |  | - 5 |  | - 6 |  |
| --- | --- | --- | --- | --- | --- | --- | --- | --- | --- | --- | --- |

1. The digital tool was presented in a user-friendly and easy-to-use environment

| - 1 |  | - 2 |  | - 3 |  | - 4 |  | - 5 |  | - 6 |  |
| --- | --- | --- | --- | --- | --- | --- | --- | --- | --- | --- | --- |

1. I used the app frequently

| - 1 |  | - 2 |  | - 3 |  | - 4 |  | - 5 |  | - 6 |  |
| --- | --- | --- | --- | --- | --- | --- | --- | --- | --- | --- | --- |

1. The educational resource was effective in helping me learn to determine the age of equines

| - 1 |  | - 2 |  | - 3 |  | - 4 |  | - 5 |  | - 6 |  |
| --- | --- | --- | --- | --- | --- | --- | --- | --- | --- | --- | --- |

1. It was effective in visually identifying wear stages

| - 1 |  | - 2 |  | - 3 |  | - 4 |  | - 5 |  | - 6 |  |
| --- | --- | --- | --- | --- | --- | --- | --- | --- | --- | --- | --- |

1. It was effective in visually identifying the type of dentition

| - 1 |  | - 2 |  | - 3 |  | - 4 |  | - 5 |  | - 6 |  |
| --- | --- | --- | --- | --- | --- | --- | --- | --- | --- | --- | --- |
